# Multiple Sclerosis as a Syndrome—Implications for Future Management

**DOI:** 10.3389/fneur.2020.00784

**Published:** 2020-08-28

**Authors:** Christopher M. Dwyer, Linda Thien-Trang Nguyen, Luke M. Healy, Ranjan Dutta, Samuel Ludwin, Jack Antel, Michele D. Binder, Trevor J. Kilpatrick

**Affiliations:** ^1^Florey Institute of Neuroscience and Mental Health, Florey Department, The University of Melbourne, Parkville, VIC, Australia; ^2^Department of Neurology, Royal Melbourne Hospital, Parkville, VIC, Australia; ^3^Neuroimmunology Unit, Montreal Neurological Institute, Department of Neurology and Neurosurgery, McGill University, Montreal, QC, Canada; ^4^Department of Neurosciences, Cleveland Clinic Lerner College of Medicine, Case Western Reserve University, Cleveland, OH, United States; ^5^Florey Institute of Neuroscience and Mental Health, Parkville, VIC, Australia; ^6^Department of Anatomy and Neuroscience, University of Melbourne, Parkville, VIC, Australia

**Keywords:** multiple sclerosis, syndrome, demyelination, innate immunity, pathophysiology

## Abstract

We propose that multiple sclerosis (MS) is best characterized as a syndrome rather than a single disease because different pathogenetic mechanisms can result in the constellation of symptoms and signs by which MS is clinically characterized. We describe several cellular mechanisms that could generate inflammatory demyelination through disruption of homeostatic interactions between immune and neural cells. We illustrate that genomics is important in identifying phenocopies, in particular for primary progressive MS. We posit that molecular profiling, rather than traditional clinical phenotyping, will facilitate meaningful patient stratification, as illustrated by interactions between HLA and a regulator of homeostatic phagocytosis, MERTK. We envisage a personalized approach to MS management where genetic, molecular, and cellular information guides management.

## Multiple Sclerosis—A Single Disease Or A SYNDROME?

Multiple sclerosis is a complex autoimmune disease of the central nervous system (CNS), characterized pathologically by inflammatory demyelination (1). The diagnosis of MS is standardized using an array of clinical, imaging, and laboratory measures, most recently described in the 2017 revised McDonald criteria (2). These require evidence of inflammatory activity within the CNS with dissemination in time and space. Temporal dissemination is not required if isolated intrathecal synthesis of oligoclonal proteins is identified. These criteria reflect a consensus based on the synthesis of clinical and paraclinical data, agnostic to the pathogenetic mechanism(s) driving neuroinflammation. We propose that this clinical phenotype of MS will be increasingly viewed as a syndrome—rather than a single disease state—because these diagnostic elements can be generated by varying pathogenetic mechanisms.

There is precedent to support this contention. Neuromyelitis optica (NMO) presents with inflammatory demyelination of the optic nerves and spinal cord and was previously considered an MS variant. We now recognize NMO is distinct, driven by unique autoantibodies (3, 4). Compellingly, NMO is now more appropriately referred to as neuromyelitis optica spectrum disorder (NMOSD), reflecting that the condition can be triggered in multiple ways, for example, by antibodies targeting aquaporin-4 expressed on astrocytes or, alternatively, by antibodies directed against the oligodendrocyte-specific myelin oligodendrocyte glycoprotein (5, 6). This demonstrates that common clinical presentations sometimes unwittingly group phenocopies, reflecting more than one pathogenic mechanism.

The concept that MS is heterogeneous was previously championed by Lucchinetti and Lassmann who described four distinct histological patterns of acute MS (7). They posited that MS was a disease of varying etiology and pathogenesis and suggested that therapy might be tailored to the individual on this basis. Unfortunately, no convincing biomarkers have emerged to enable reliable and valid clinical stratification according to these parameters and the idea was not uniformly accepted (8). However, recent advances in identifying genomic, immuno-biological, and environmental contributors to MS pathogenesis call for reexamination of this issue. For example, Trapp et al. recently described MS histopathology that demonstrated neurodegeneration with cortical and spinal myelin loss but without cerebral white-matter demyelination, so-called myelocortical MS (MCMS). While clinical and MRI findings in MCMS and typical MS are indistinguishable, it is possible that the pathophysiologies driving these presentations are markedly different, requiring different therapeutic approaches (9).

Genetic factors and environmental exposures contribute to MS susceptibility, although, consistent with a syndrome-based hypothesis, no algorithm that meaningfully quantifies individual risk has been identified (10). Caucasians heterozygous for HLA-DRB1^*^1501 within the major histocompatibility complex (MHC) have an odds ratio for developing MS of ~3.0, representing the strongest susceptibility allele for MS (11). Over 200 other susceptibility loci have been identified via genome-wide association studies (GWAS), but each conveys an odds ratio of 1.2 or less (12). Prior infection by the Epstein–Barr virus (EBV) appears necessary, but not sufficient, to cause MS (13). Other environmental factors that contribute to risk include smoking, hypovitaminosis D, and obesity (14, 15). How these various factors contribute at an individual level to pathogenesis and clinical phenotype remains unknown.

## New Approaches to Assessing the Pathophysiology Of MS

How can we interrogate the pathogenesis of MS more effectively? The neurological signs and symptoms of MS are non-parametric traits and attempts to use them to meaningfully sub-stratify the disease have not been helpful (16). The alternative, as proven successful in other contexts, is to classify using cellular and molecular profiling (17). If successful, such an approach would have major implications for both prognostication and therapeutic targeting in MS.

Next-generation sequencing of MS pedigrees, although hampered by incomplete penetrance and limited due to pedigree scarcity, could also offer new insights (18). Enticingly, mutations in genes implicated in cholesterol metabolism and oxysterol synthesis have been recently reported in MS pedigrees (19). It will be important to determine whether these mutations are also more prevalent in sporadic MS, similar to the genetic links identified between pedigree-based and sporadic Parkinson's disease (20).

A subset of progressive MS patients demonstrate mutations pathogenic for other neurological conditions (21). Certain of the identified genes could be implicated in MS pathophysiology through, for example, influences upon myelin composition or microglial development (22–24). It will be important to understand what factors, in addition to these mutations, are necessary to induce the inflammation and neurodegeneration implicated in MS progression.

If MS is a syndrome, are there ways that the condition can be classified holistically beyond clinical definitions? The traditional view that MS reflects T-effector activity induced by one or more autoantigens provides such a framework. This perspective has been very recently advanced by Martin et al. who identified auto-proliferative T-effector clones in MS patients during remission rather than relapse, as might have been presumed *a priori* (25). T-effector cell reactivity was induced in a HLA-DRB1^*^1501-positive MS patient by presentation of a peptide derivative of Ras guanine nucleotide-releasing protein 2 (RASGRP2), a calcium sensor expressed within the CNS. Whether this pathogenic mechanism applies to the broader MS population and whether RASGRP2 is an initiator of disease or a reflection of epitope spreading awaits clarification.

The influence that specific molecules exert upon adaptive immunity and disease pathogenesis can be contextual. For example, we discovered that the co-stimulatory molecule CD40 is a risk gene for MS (26). We found the responsible single-nucleotide polymorphism (SNP) is in the Kozak consensus sequence, which drives expression of the CD40 protein and that, paradoxically, this genetic variation leads to reduced CD40 in B lymphocytes and other mononuclear cells (27). Intriguingly, this SNP also conveys susceptibility to Graves' disease and rheumatoid arthritis but susceptibility in these diseases rests with the high-expressing allele. Clearly, there are subtleties in molecular signaling that influence autoimmune disease phenotype in ways we are yet to understand. This perspective, although challenging, is not iconoclastic; single antigens can induce either tolerance or pathogenic T-effector cell-induced autoimmunity depending on the route of autoantigen administration (28), and the same myelin basic protein-derived peptide can induce anti- and pro-inflammatory responses in T cells derived from MS patients (29).

The innate immune system also plays a central role in MS pathogenesis and potentially in MS initiation as recently reported by the International Multiple Sclerosis Genetics Consortium (30). This builds on earlier work, demonstrating that in a subset of pathological specimens oligodendrocyte injury and microglial activation appear to precede T-lymphocyte infiltration (31). The innate immune system is composite; it includes professional antigen-presenting cells (APCs) such as dendritic cells and mononuclear cells such as microglia and macrophages that can present antigen and, in a context-dependent manner, either promote or inhibit inflammation via cytokine production (32). Mononuclear cells can also phagocytose pathogens, cellular debris, and even living cells (33). Intimate associations between macrophages and neural cells occur in MS, suggesting innate immune cells could disrupt axons and myelin (34). Conversely, innate immune activation and phagocytosis of myelin debris appear necessary for oligodendrocyte differentiation and remyelination (35). Recent work has also identified key differences between peripheral macrophages and central microglia with respect to expression profiling and functional activity, with microglia being more likely to assume an anti-inflammatory, reparative phenotype (36). In a context-dependent manner, microglia can also assume either a pro- or anti-inflammatory function (37).

## Seeking an Overarching Model of Central Inflammatory Demyelination

How can we assimilate these various perspectives concerning disease pathogenesis? An overarching hypothesis of chronic inflammatory demyelination posits a breakdown in homeostatic interactions between a target cell (presumably the oligodendrocyte) and the innate and adaptive immune systems, recognizing some trafficking of lymphocytes occurs through the healthy CNS (38). This breakdown could be orchestrated by several mechanisms.

**Figure 1:**
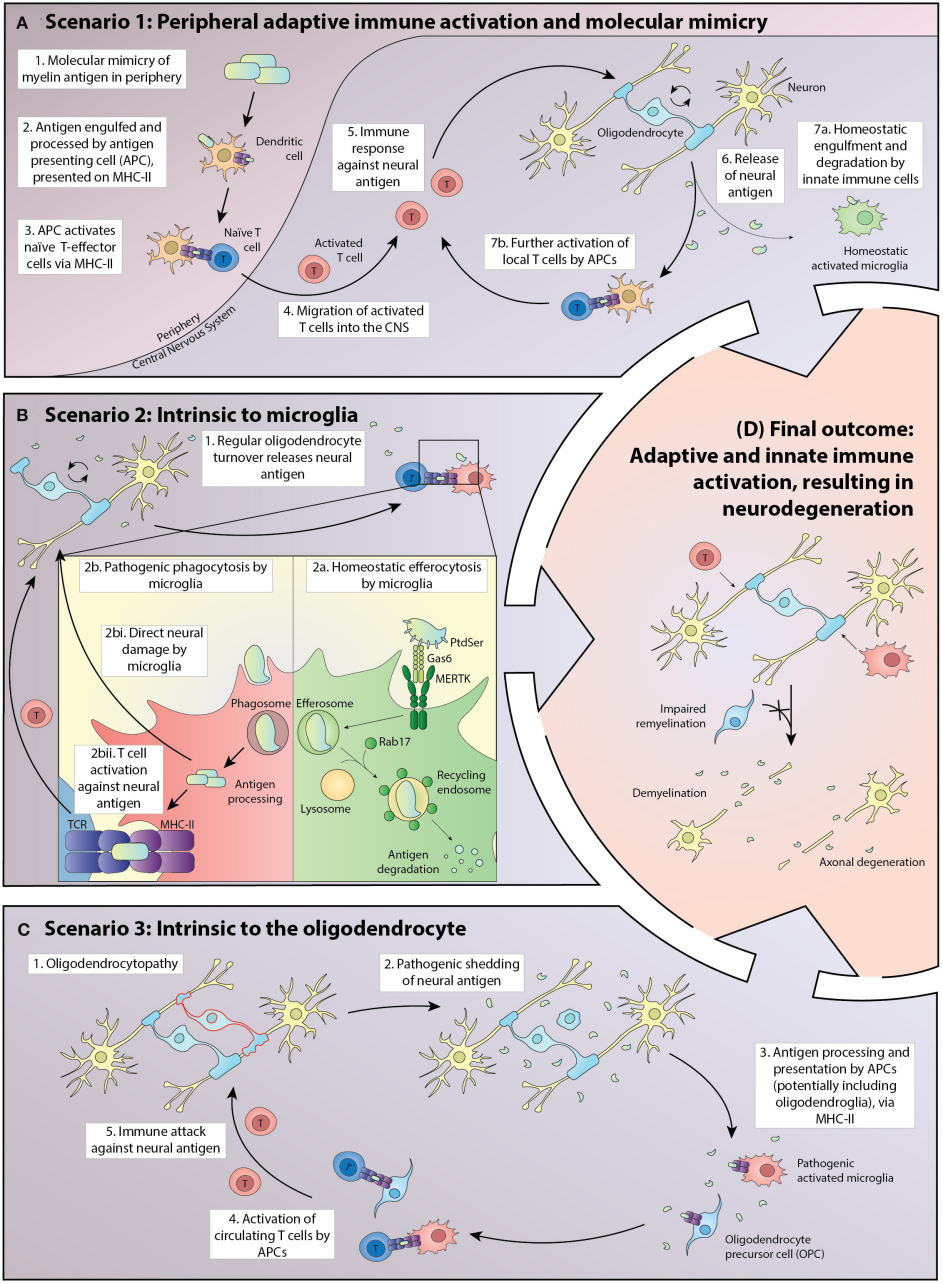
Potential scenarios driving the pathogenesis of MS in which homeostatic interactions between oligodendroglia and the adaptive and innate immune systems break down, leading to inflammatory demyelination, and eventually to neurodegeneration. **(A)** In Scenario 1, exogenous antigen is processed in peripheral lymphoid tissue and presented to naïve T cells via MHC Class II. A small proportion of the activated T-effector cells traffic through the CNS where some turnover of neural cell macromolecules, including myelin proteins, occurs. Those molecules are either degraded by the innate immune system or are presented at a basal level by antigen-presenting cells (APCs), principally microglia but potentially also oligodendrocytes and their progenitors. Where the exogenous antigen exhibits molecular mimicry to a neural antigen, there is the potential for further activation of inflammatory T cells within the CNS. This leads to a positive feedback loop in which the T-effector cells initiate CNS damage, resulting in increased presentation of neural antigen by APCs and accelerated proliferation and activation of T-effectors locally. **(B)** In Scenario 2, the processing of neural antigens within APCs becomes corrupted, with the balance shifting from efferocytosis in which molecules are digested, to one in which peptides are presented via MHC class II. Homeostatic mechanisms potentially corrupted include the presentation of phospholipids to the APC by a limited number of cell surface receptors, including MERTK and its ligands proteins and Gas6. MERTK signaling primes the cell to efferocytosis as does the intracellular protein Rab7, which activates lysosomal induced molecular degradation rather than antigen presentation. In this scenario, microglia both have an initial pathogenic role and activate naïve T cells that normally transit through the CNS and to draining cervical lymph nodes in an immune surveillance role, but with potential for reactivity against neural antigens once homeostasis is disrupted. **(C)** In Scenario 3, pathology intrinsic to oligodendrocytes results in the presentation of neuronal antigens by APCs, including microglia and potentially oligodendroglia. The initial pathology is an oligodendrocytopathy with acceleration of disease driven via activation of microglia and eventually of circulating T cells. **(D)** Independent of the initiating event, breakdown in homeostatic interactions within the CNS ultimately leads to relatively stereotyped pathology characterized by oligodendrocyte loss, demyelination, and axonal degeneration.

A favored pathology (Figure 1, Scenario 1) embraces the classical perspective of an adaptive immune system inappropriately induced by antigenic stimuli to target neural cells, potentially via molecular mimicry. Exogenous or CNS antigen is first presented in regional lymphoid tissue adjacent to the entry site, typically in deep cervical lymph nodes where either tolerance or auto-reactivity can be induced, depending on the nature of the antigen and its immune processing prior to presentation (39). Where auto-reactivity is induced, full activation of the lymphocyte population requires the expression of appropriately configured self-antigen by perivascular mononuclear cells within the CNS (40).

Disturbance in the processing of oligodendrocyte antigens normally cleared by the innate immune system (Figure 1, Scenario 2) could represent another mechanism by which homeostasis is disrupted. Sculpting of the oligodendrocyte membrane is an active, normal developmental process and potentially extends throughout life (41). Insults either directly or indirectly targeting microglia could catalyze a maladaptive response leading to spurious Toll-like receptor (TLR) activation, as previously demonstrated in systemic lupus erythematosus (SLE) (42). Aberrant TLR activation could corrupt the processing and presentation of oligodendrocytic antigens. Cytokine and reactive oxygen species produced by activated microglia also could be an important early pathogenic event, independent of adaptive immune system activation (43). This pathophysiology would accordingly bypass the presentation of phospholipids to the APC by pro-homeostatic mechanisms, including MERTK and its ligands Proteins and Gas6. MERTK signaling primes the cell to efferocytosis as does the intracellular protein Rab7, which activates lysosomal induced molecular degradation rather than antigen presentation.

A third mechanism (Figure 1, Scenario 3) could involve pathology intrinsic to the oligodendrocyte, possibly established developmentally but most commonly revealed in adult life. A long prodromal period of this nature is typical of other neurodegenerative diseases and is sometimes identified in MS following detection of asymptomatic neuroinflammatory lesions on magnetic resonance imaging undertaken for unrelated reasons (44). The oligodendrocyte is a metabolically active cell with ongoing turnover of myelin throughout life (45). Corruption of the nature or kinetics of oligodendrocyte metabolism, leading to pathogenic shedding of antigen, could represent the first insult predisposing to inflammatory demyelination (46). The concept that oligodendroglia are active immunomodulators in MS has also been recently further championed by Castelo-Branco et al., having identified disease-specific oligodendroglia that express the major histocompatibility complex (MHC) in the MS brain (47). Oligodendroglia that participate in antigen presentation were also recently described (48).

Irrespective of the disease initiator, in established MS oligodendrocytes are targeted and the adaptive and innate immune systems are activated. Corruption of oligodendrocyte viability leads to antigen processing within the CNS, with macrophages and microglia expressing MHC class II and costimulatory molecules (49–51). In this context, a subset of the repertoire of lymphocytes that normally traffic through the CNS can become activated, with some of the cells exposed to self-antigen trafficking back to the cervical lymph nodes (52) to accelerate an adaptive immune response systemically, a process which plays out in all our described scenarios (Figure 1) (53–55). Regional stratification of adaptive immune targeting has been reported in animal models, with myelin-reactive T cells observed to infiltrate white matter while T-cells recognizing the neuronal protein synuclein target gray matter, a phenomenon that could contribute to the duality of MS immunopathology, with cortical destruction by synuclein-reactive T cells representing a mediator of neurodegeneration (56).

### Striking a Balance Between Pro- and Anti-inflammatory Phagocytosis

Homeostasis can be reestablished by innate immune cells when they engulf and digest apoptotic cells and necrotic debris without presenting self-antigens via the MHC (57). In contrast, the innate immune system can present antigen, induce inflammation, and recruit the adaptive immune system, which is an appropriate response to pathogens but, when corrupted, promotes autoimmunity (58). In MS, the disruption of homeostasis enables antigens released from the oligodendrocyte or molecules expressed at paranodes to be presented via innate immune cells to pathogenic T-effector cells.

Whether homeostasis is restored, or autoimmunity promoted, will be dependent upon the molecular environment. Homeostatic phagocytosis by innate immune cells is facilitated by “eat me” signals including phosphatidylserine (PtdSer) and other phospholipids actively “flipped” onto the external surface of the cell membrane of apoptotic cells (59). These lipids are also found in myelin debris. Several secreted and cell surface receptors expressed by phagocytes recognize these lipids and respond to the “eat me” signal. One of these recognition molecules is the receptor tyrosine kinase, MERTK, which is encoded by a susceptibility gene for MS (60, 61). Ligands for MERTK, namely, Protein S (ProS) and growth arrest-specific 6 (Gas6), act as opsonins or molecular bridges between PtdSer presented by apoptotic cells and MERTK expressing phagocytes. The resultant signaling within the phagocytic cell influences how the engulfed proteins are processed. Under the influence of MERTK, these proteins potentially bypass the phagosomes that would otherwise facilitate antigen presentation by MHC and predispose to autoimmunity (Figure 1, Scenario 2) (62). Interestingly, in SLE, increased soluble MERTK (which negates the anti-inflammatory effect of membrane-bound MERTK signaling by APCs) is associated with increased autoantibody production, a marker of disease activity (63).

### TAM Receptors as Key Regulators of MS Pathogenesis

MERTK is a member of the TAM (TYRO3, AXL, and MERTK) family of receptor tyrosine kinases. The TAMs are part of a dynamic, compensatory system that responds to inflammatory insults. TAM receptor expression on innate immune cells is upregulated in response to phagocytosis, via activation of the retinoic acid pathway (64).

We identified *MERTK* as both a susceptibility gene for MS and an expression quantitative trait locus (eQTL) within innate immune cells in the peripheral blood (60). The predominant susceptibility alleles of *MERTK* for a majority of the MS population drive high expression of MERTK in innate immune cells. However, for people homozygous for HLA-DRB1^*^1501, itself a major susceptibility gene for MS, there is enhanced susceptibility and severity of the disease among those homozygous for low expressing *MERTK* alleles. This stratification of risk and severity based on HLA-DRB1^*^1501 status suggests the contextual influence of MERTK is via antigen presentation. This provides further support for the concept of disease heterogeneity and the need for targeted molecular phenotyping.

#### Non-HLA-Mediated Effects

In the majority of MS cases, the direct contribution of adaptive immunity from the peripheral blood diminishes with time (65). Concurrently, immune therapies that target adaptive immune responses lose efficacy (66). Whether disease pathogenesis in progressive disease reflects a shift to intrinsic neurodegeneration, persistent activity of pathogenic adaptive immune cells behind an intact blood–brain barrier or corruption of innate immune system activity that subsequently leads to neurodegeneration, remains unknown.

MERTK expressed by microglia and by infiltrating macrophages could promote the efficient removal of debris, once MS is initiated. There are precedents for this function. For example, MERTK is important in preventing retinitis pigmentosa by abrogating the accumulation of debris from photoreceptor cells (67). The level of expression of MERTK on human microglia strongly correlates with the capacity of these cells to engulf myelin debris, of importance as the ability to clear myelin from the damaged CNS is essential for myelin repair (68, 69). Our preliminary analysis of pathological material indicates that for patients with secondary progressive MS, there is robust expression of MERTK in periplaque microglia, although the level of expression varies between specimens (Ranjan Dutta, personal communication), indicating that this phagocytic role is likely to be relevant to MS but of variable importance between individuals.

### Intrinsic Oligodendrocytopathies in MS Pathogenesis

Demyelinating diseases can be acquired or inherited. Among inherited leukodystrophies, cerebral adrenoleukodystrophy (X-ALD) is exceptional for associated T cell-mediated inflammation. CSF examination of patients with X-ALD reveals oligoclonal T-cell expansion, as seen in MS (70). X-ALD is caused by mutations in the X-linked *ABCD1* gene that encodes a peroxisomal transporter. The maintenance of myelinated axons requires continual turnover of myelin membranes and oligodendroglial peroxisomes play an intimate role in lipid metabolism in that context. Perturbations in the removal of lipid debris by oligodendrocytes could facilitate an inflammatory cascade in which local microglia/macrophages become autoreactive. Specifically, a failure to metabolize eicosanoids, which are potent mediators of inflammation and are increased in a range of neurodegenerative conditions, could result from intrinsic peroxisome dysfunction, leading to the phenotype seen in X-ALD but also in a subset of MS patients (31, 46, 71).

## The Future: Using Genetic, Molecular, and Cellular Findings to Guide Clinical Management

We believe it is essential to adopt a precision medicine, biomarker-based approach that looks beyond a singular focus on adaptive immune responses to further benefit patients with MS, in particular those at risk of progressive disease. Understanding what drives MS progression, and recognizing that this may be heterogeneous, is essential for the efficient deployment of novel therapeutic interventions.

Our preliminary findings suggest that at least 10% of people currently diagnosed with primary progressive MS could harbor mutations for other genetic neurodegenerative diseases (21). This is particularly germane now that anti-CD20 immunotherapy is reported to benefit a subset of patients with primary progressive disease (72). Clearly, the expense and risks of this therapy should be restricted to people who demonstrably have MS rather than phenocopies. Moreover, once patients begin treatment with therapies specific to progressive MS their subsequent disease course must be monitored with valid, reliable, and responsive biomarkers to establish—at the individual level—that benefit is being achieved in order to justify incumbent risks. Serial assessment of phosphorylated neurofilament light chain levels in either blood or CSF is one biomarker with potential in this regard (73).

In addition, the MS clinic of the future will need to embrace genomics to detect phenocopies and substratify the disease in meaningful ways. For example, our data already indicate that a key modulator of innate immune function, MERTK, influences not only susceptibility but also potentially disease severity but in an HLA-dependent manner (60, 61). If next-generation therapies targeting MERTK and other innate immune modulators become available, it will be important to determine, *a priori*, which patients will benefit; for example, the potentiation of MERTK signaling for therapeutic benefit could be predicated on HLA-DR15 homozygosity to enhance a tolerogenic phenotype (60). The variable expression of MERTK by microglia in autopsy specimens indicates the need to understand whether variance in microglial activity, as can be determined by PET-based imaging (74), correlates with clinical phenotype. Such information could inform future translational research, by identifying patients who would be most likely to benefit from therapies targeting microglial activity.

Finally, although the promise of regenerative therapy has garnered significant attention, we believe that most impactful therapeutic advances in MS will involve the prevention of CNS damage (66). Only by understanding the diversity of pathogenetic mechanisms driving neuroinflammation and degeneration in MS-like presentations will we achieve this goal.

## Author Contributions

CD and TK wrote the manuscript. LN produced the figure. LN, LH, RD, SL, JA, and MB provided conceptual input and edited the manuscript. All authors read and approved the final manuscript.

## Conflict of Interest

The authors declare that the research was conducted in the absence of any commercial or financial relationships that could be construed as a potential conflict of interest.
